# Effect of 6p21 region on lung function is modified by smoking: a genome-wide interaction study

**DOI:** 10.1038/s41598-020-70092-0

**Published:** 2020-08-04

**Authors:** Boram Park, Jaehoon An, Wonji Kim, Hae Yeon Kang, Sang Baek Koh, Bermseok Oh, Keum Ji Jung, Sun Ha Jee, Woo Jin Kim, Michael H. Cho, Edwin K. Silverman, Taesung Park, Sungho Won

**Affiliations:** 1 Department of Public Health Sciences, Seoul National University, Seoul, South Korea; 2 Interdisciplinary Program of Bioinformatics, Seoul National University, Seoul, South Korea; 3 Department of Internal Medicine, Healthcare Research Institute, Seoul National University Hospital Healthcare System Gangnam Center, Seoul, South Korea; 4 Department of Preventive Medicine, Yonsei University Wonju College of Medicine, Wonju, South Korea; 5 Department of Biochemistry and Molecular Biology, School of Medicine, Kyung Hee University, Seoul, South Korea; 6 Institute for Health Promotion, Graduate School of Public Health, Yonsei University, Seoul, South Korea; 7 Department of Internal Medicine and Environmental Health Center, Kangwon National University Hospital, School of Medicine, Kangwon University, Chuncheon, South Korea; 8 Channing Division of Network Medicine, Department of Medicine, Brigham and Women’s Hospital and Harvard Medical School, Boston, MA USA; 9 Division of Pulmonary and Critical Care Medicine, Department of Medicine, Brigham and Women’s Hospital, Harvard Medical School, Boston, MA USA; 10 Department of Statistics, Seoul National University, Seoul, South Korea; 11 Institute of Health and Environment, Seoul National University, Seoul, South Korea

**Keywords:** Biomarkers, Diseases, Genetics, Genetic interaction, Genetic markers

## Abstract

Smoking is a major risk factor for chronic obstructive pulmonary disease (COPD); however, more than 25% of COPD patients are non-smokers, and gene-by-smoking interactions are expected to affect COPD onset. We aimed to identify the common genetic variants interacting with pack-years of smoking on FEV_1_/FVC ratios in individuals with normal lung function. A genome-wide interaction study (GWIS) on FEV_1_/FVC was performed for individuals with FEV_1_/FVC ratio ≥ 70 in the Korea Associated Resource cohort data, and significant SNPs were validated using data from two other Korean cohorts. The GWIS revealed that rs10947231 and rs8192575 met genome-wide significant levels; For ${\text{H}}_{0} :\beta_{SNP} = \beta_{SNP*pack - years} = 0{\text{ vs H}}_{1} : not {\text{H}}_{0} ,$ the likelihood ratio (LR) test was conducted, and its *P* values, *P*_LR_, for rs10947231 and rs8192575 were 2.23 × 10^–12^ and 1.18 × 10^–8^, respectively. Interaction between rs8192575 and smoking is significantly replicated with two additional data (*P*_INT_ = 0.0454, 0.0131). Expression quantitative trait loci, topologically associated domains, and PrediXcan analyses revealed that rs8192575 is significantly associated with *AGER* expression. SNPs on the 6p21 region are associated with FEV_1_/FVC, and the effect of smoking on FEV_1_/FVC differs among the associated genotypes.

## Introduction

Chronic obstructive pulmonary disease (COPD) is a common respiratory disease with high worldwide morbidity and mortality^1^, characterized by progressive airflow obstruction^2^. Although cigarette smoking is the major environmental risk factor for COPD, multiple factors can contribute to this disease, including air pollution, infection, and asthma^1,3^. However, sensitivity to smoking differs among individuals, and only a minority of smokers develop COPD^4,5^, highlighting the potential importance of genetic architecture. Severe ${\mathrm{\alpha }}_{1}$-antitrypsin (AAT) deficiency is the best known genetic risk factor for COPD^6^, and genome-wide association studies (GWAS) have identified multiple promising candidate genes for COPD, including *FAM13A*, *HTR4*, *RIN3*, *HHIP*, *ADAM19*, *CHRNA3/5*, *AGER*, and *EEFSEC*^6–8^. Estimated heritabilities in family aggregation studies are typically 30%^9^. Notwithstanding the importance of genetic architecture, AAT deficiency occurs in only 1 ~ 2% of COPD patients^5^ and the pathological role of most COPD candidate genes is unknown^10^. Although COPD results from genetic and environmental factors,limited information is available regarding the genetic factors that actually contribute to COPD, and gene-environment interactions, except for AAT deficiency, have been difficult to identify^11^.

Nevertheless, COPD is known to be strongly influenced by cigarette smoking and multiple genetic variants, and recent studies have reported gene-environment interactions. For instance, Aschard et al.^12^ calculated the genetic risk scores for 26 genome-wide significant single nucleotide polymorphisms (SNPs), and reported significant interactions between genetic risk scores and smoking. In addition, both Hancock et al.^13^ and Park et al.^14^ hypothesized the existence of SNP-by-smoking interactions,the first performed a genome-wide interaction study on pulmonary function, modelling the primary effects of single SNPs and their interactions, while the latter reported *SOX9*-by-smoking interactions. However, there is heteroscedasticity in pulmonary function between smokers and non-smokers. Furthermore, smoking has a nonlinear effect on pulmonary function^15^, and the heterogeneity of this association has complicated SNP-by-smoking interaction analyses, thus limiting the number of identified interactions.

COPD is a progressive diseases that can be prevented but is irreversible^16^, so the most effective way to prevent COPD is primary prevention. Primary prevention involves two concepts: (1) to keep healthy individuals consistently healthy (health promotion), and (2) to prevent the onset or exacerbation of the diseases (disease prevention)^17^. Therefore, it is important to maintain healthy lung function, and we focus on the genetic and environmental factors in people with healthy lung function. In our previous reports^14^, we analyzed whole individuals on FEV_1_, and thus in this article, we aimed to identify genetic variants interacting with smoking, using spirometry measurements of FEV_1_/FVC ratios from individuals with FEV_1_/FVC ≥ 70 in the Korea Associated Resource (KARE), as this measurement determines the presence of airflow limitation and obstructive lung diseases such as COPD. We assessed significant SNPs on our genome-wide interaction study (GWIS) using Gene-Environment of Interaction and phenotype (GENIE) and Atherosclerosis Risk of a Rural Area in Korean General population (ARIRANG) data. We focused on potential SNP-by-smoking interactions considering only the SNPs that reached overall genome-wide significance levels.

## Materials and methods

The KARE cohort was used for the discovery analysis in GWIS; GENIE and ARIRANG cohorts were included in validation analysis. Only participants with FEV_1_/FVC ratio ≥ 70 were included.

### Discovery analysis using KARE

**Figure 1:**
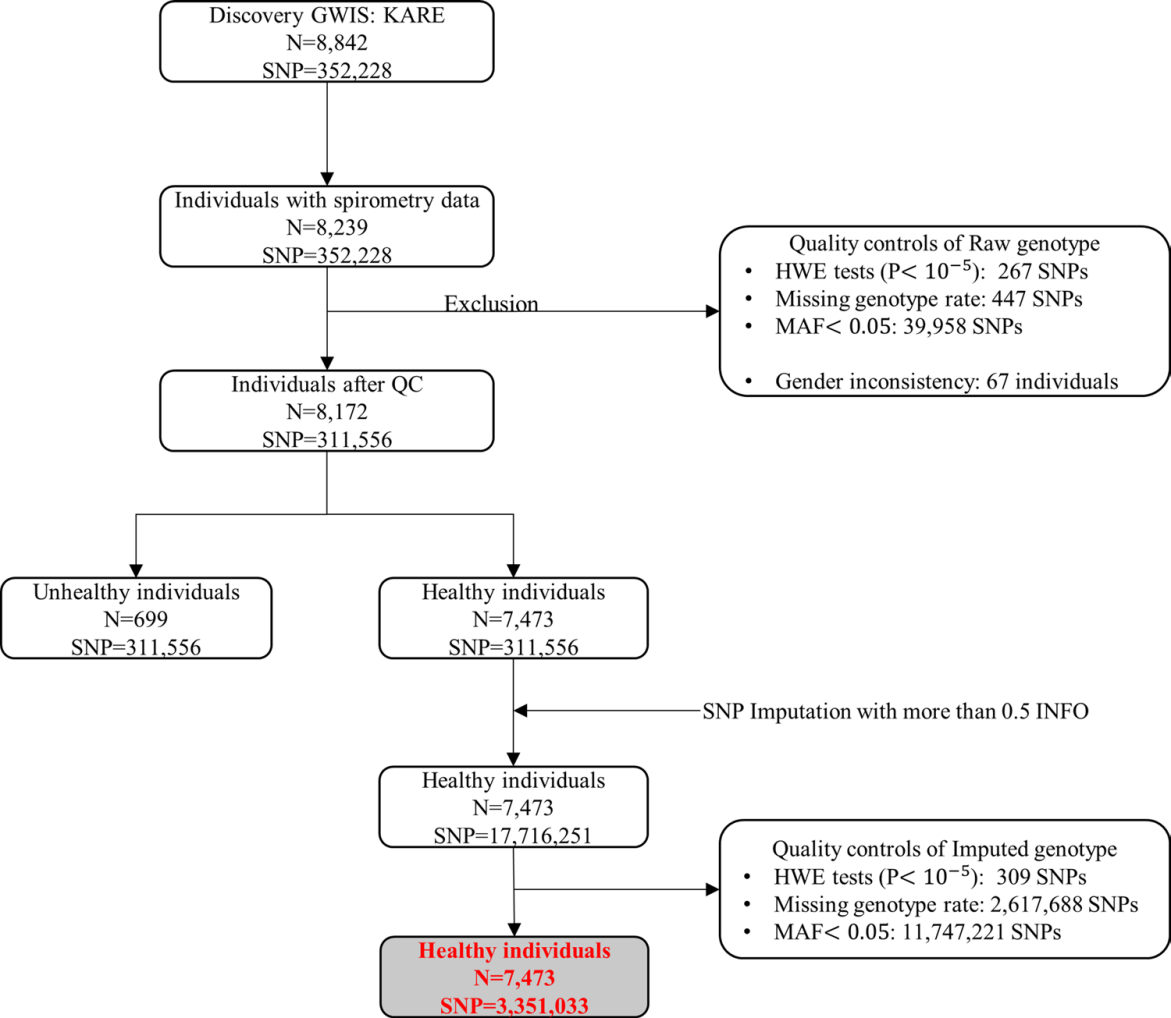
Flow diagram for KARE cohort Fig. 1 explains how the individuals and SNPs were included and excluded. After quality controls and imputations, finally 7473 healthy individuals and 3,351,033 SNPs were used for analyses.

The KARE project was initiated in 2007 for a large-scale GWAS, and participants constituting the independent Ansan and Ansung cohorts were included in the Korean Genome Epidemiology Study (KoGES)^18^. KoGES involved longitudinal prospective studies on 5018 participants in Ansung and 5020 participants aged 40–60 years in the Ansan area. KARE genotype data were obtained using the Affymetrix Genome-Wide Human SNP array 5.0^18^, and quality control analyses were performed,8172 participants underwent spirometry analysis, and their smoking history was recorded (Fig. 1). Smoking history was obtained from questionnaires, and pack-years of smoking was considered for analyzing SNP-by-smoking interactions. Among the 8172 participants, 7473 participants showed FEV_1_/FVC ratio ≥ 70; 4768 were non-smokers, 1140 were former smokers, and 1565 were current smokers.

### Validation analyses using GENIE and ARIRANG data

The GENIE cohort comprised 7999 participants agreeing to provide blood samples and to participate in genetic studies that had visited Seoul National University Gangnam Center in 2014.^19^ Participants underwent genotype analysis using the Affymetrix Axiom KORV1.1–96 Array^20^ and genotype quality control (QC) was performed. The 4413 participants with FEV_1_/FVC ratio ≥ 70, age > 40 years, available spirometric data, and smoking history were included in the association analysis. Based on questionnaires, 2520 individuals were non-smokers, 1380 were former smokers, and 513 were current smokers. From this dataset, the 2520 non-smokers and 513 current smokers were included in the GWIS validation study.

ARIRANG is an ongoing study on cardiovascular and metabolic risk factors, and participants aged 40–70 years are part of the KoGES study in rural Wonju and Pyengchang^21^. The ARIRANG genotype data were obtained using the Affymetrix Genome-Wide Human SNP array 6.0, and genotype QC was performed. Spirometry data and smoking history were available for 513 participants with FEV_1_/FVC ratio ≥ 70. Based on questionnaires, 369 individuals were non-smokers, 65 were former smokers, and 79 were current smokers. All participants were included in the GWIS.

### Quality control

For the discovery GWIS with KARE data, as well as for the validation analyses using GENIE and ARIRANG, the QC of SNPs and subjects was conducted using PLINK^22^ and oneTOOL^23^. We excluded SNPs with *P* values on the Hardy–Weinberg equilibrium (HWE) analysis < 10^–5^, minor allele frequencies (MAFs) < 0.05, and genotype call rates < 95%. Furthermore, we excluded subjects with missing genotype call rates > 5% or sex-based inconsistencies. After QC, 311,556 SNPs and 7473 participants with FEV_1_/FVC ratio ≥ 70 were included for whole-genome imputation.

### Genotype imputation

For GWIS with KARE data, whole-genome imputation was performed using SHAPEIT2 and IMPUTE2 for pre-phasing data and genotype imputations. The 1000 Genomes Phase 3 was used as the reference panel. To maintain imputation quality, the estimated imputation accuracy for imputed SNPs was evaluated using the INFO metric, and any imputed SNPs with INFO < 0.5 were eliminated. The standard QC procedure was also applied for imputed SNPs, and 3,351,033 SNPs from 7473 participants were used for the GWIS discovery study (Fig. 1).

For the validation analyses, genotypes comprising the most significant SNPs were not originally genotyped, and target imputation was conducted. Target imputation for regions containing significantly associated SNPs was performed using IMPUTE2 with a buffer size of 5 million bp for each target SNP.

### GWIS with KARE data

The GWIS discovery study of the FEV_1_/FVC ratios with KARE data was conducted for healthy individuals. We found that the most of our samples do not have any COPD, and the number of COPD patients is only 699. Genetic association analysis needs large sample sizes^24^, and thus we decided to focus on healthy individuals with FEV_1_/FVC ratio ≥ 70. To handle heteroscedasticity in pulmonary function between non-smokers and smokers, the weighted least squares regression was used with inverse variance weights according to smoking status (non-smoker or smoker). To assess the appropriateness of the weighted least analysis, we compared its Akaike Information Criterion with the linear regression coefficient, and found that the weighted least squares regression had better fit. Age, sex, BMI, age × sex, and pack-years of smoking were included as covariates. Principal component (PC) scores were estimated from the genetic relationship matrix, and 10 PC scores corresponding to the 10 largest eigenvalues were included as covariates to adjust the population substructure. For the GWIS between each SNP and pack-years of smoking, we fitted the following weighted least squares regression. Considering ${\mathrm{y}}_{\mathrm{j}}$ the FEV_1_/FVC values for smoking status $j$, and $j$=0 and 1, indicating non-smokers and smokers, respectively,

$$ \begin{aligned} & y_{j} \sim \beta_{0} + \beta_{1} age + \beta_{2} sex + \beta_{3} BMI + \beta_{4} age \times sex + \beta_{5} pack \;years + \mathop \sum \limits_{k = 1}^{10} \tau_{k} PC^{k} + \beta_{6} SNP \\ & \quad + \beta_{7} SNP \times pack\;years + \epsilon_{j} , where \epsilon_{j} \sim N\left( {0,w_{j} \sigma^{2} } \right). \\ \end{aligned} $$

Here, for ${w}_{1}$ and ${w}_{2}$, we estimated the residual variances from the linear regressions with only non-smokers and smokers, respectively, and the inverse of residual variances was used.

To identify SNPs interacting with pack-years of smoking on FEV_1_/FVC values, we considered ${\mathrm{H}}_{0}:{\beta }_{6}={\beta }_{7}=0$, and the hypothesis was tested by the likelihood ratio (LR) test with two degrees of freedom (DF) for healthy individuals. To adjust the multiple testing issue, the *P* values for testing ${\mathrm{H}}_{0}:{\beta }_{6}={\beta }_{7}=0$ was set to the genome-wide significant level of 5 × 10^–8^.

### Validation studies

The genome-wide significant SNPs resulting from the GWIS discovery study were replicated using the GENIE and ARIRANG datasets. For the ARIRANG dataset, similar to that performed for the GWIS discovery analysis, healthy individuals were included and the weighted least squares regression was fitted. However, for GENIE, the former smokers were excluded because they consisted of participants who had regular health check-ups and consults for health improvement, including smoking cessation, regular exercise, etc.^19^ Even a short interference of 3 min is said to significantly increase the rate of smoking cessation among smokers^25^, and this could bias the data. For the weighted least squares regression of the GENIE data, the weight was estimated using non-smokers and smokers.

A *P* value < 0.05 was set as the significance level in all analyses.

### Topologically associating domains, PrediXcan, and expression quantitative trait loci analyses

Topologically Associating Domains (TADs) are genomic regions that exhibit high levels of chromatin interactions within a region or domain, but with little or no interaction with external regions^26^. These domains are consistent across cell types and highly conserved across species, indicating that the TADs properties are strongly conserved in mammals^27^. TADs were considered to identify boundaries where causal variants can greatly influence tissue-independent function^28^. We used the web-based 3D Genome Browser^29^ to identify TADs of significant SNPs from the GWIS and to confirm interactive protein-coding genes within TADs. We selected human tissues per the hg19 assembly and explored available high-throughput chromosome conformation capture (Hi-C) data from lung tissue obtained from donor 1 (Accessing number: SRX2179252 from GEO database).

PrediXcan, a gene-based approach for identifying genes associated with the phenotype of interest^30^, imputes unobserved gene expression levels from genotypes and analyzes associations between imputed gene expression and phenotype. The imputation model for gene expression was developed for 48 different human tissues with Genotype-Tissue Expression (GTEx) V7 data. PrediXcan was used to impute gene expression of lung tissue and its association with the FEV_1_/FVC ratio.

We analyzed expression quantitative trait loci (eQTL) to investigate genetic variants associated with gene expression levels. For the eQTL analysis, we used the GTEx portal providing reference resources of genetic variation and gene regulation in diverse human tissues^31^.

### SNP-exposure independence

SNP-by-environment interactions can be significant in the absence of true SNP-by-environment interactions in cases of SNP-environment dependencies^32^. Thus, correlations between most genome-wide significant SNPs from the GWIS discovery study and smoking were assessed. As smoking variables, we considered smoking status and pack-years, which were considered independent responses. SNPs were considered covariates for both scenarios. Smoking status was either non-smoker or smoker, and logistic regression analysis was conducted. For pack-years, linear regressions were performed.

### Research ethics approval

This study complies with the scholarly and ethical conduct in research involving human participants. All study participants provided informed consent, and the study design was approved by the Institutional Review Board (IRB) at Seoul National University (IRB No. E1605/E002-003). All methods were performed in accordance with the relevant guidelines and regulations.

## Results

### GWIS of the FEV_1_/FVC ratio among the healthy individuals from the KARE cohort

**Figure 2:**
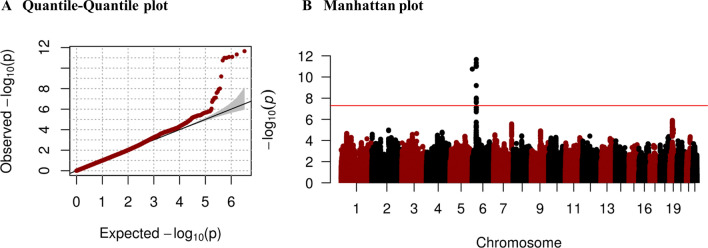
Quantile–quantile plot and Manhattan plot from GWIS with healthy individuals in KARE data (**A**) is obtained from the comparison between observed *P* values quantiles relative and expected quantiles under the uniform distribution (null hypothesis). The variance inflation factor (VIF) was 1.002, suggesting our results are free of systematic *P *value inflation. (**B**) was plotted from the logarithms of the *P *values of 3,351,033 SNPs against its physical chromosomal position. The red line represents genome-wide significance level (5 × 10^–8^), and several SNPs located at 6p21 meet this significance level. The plot was generated by software R version 3.6.1 (R Foundation for Statistical Computing; Vienna, Austria).

**Figure 3:**
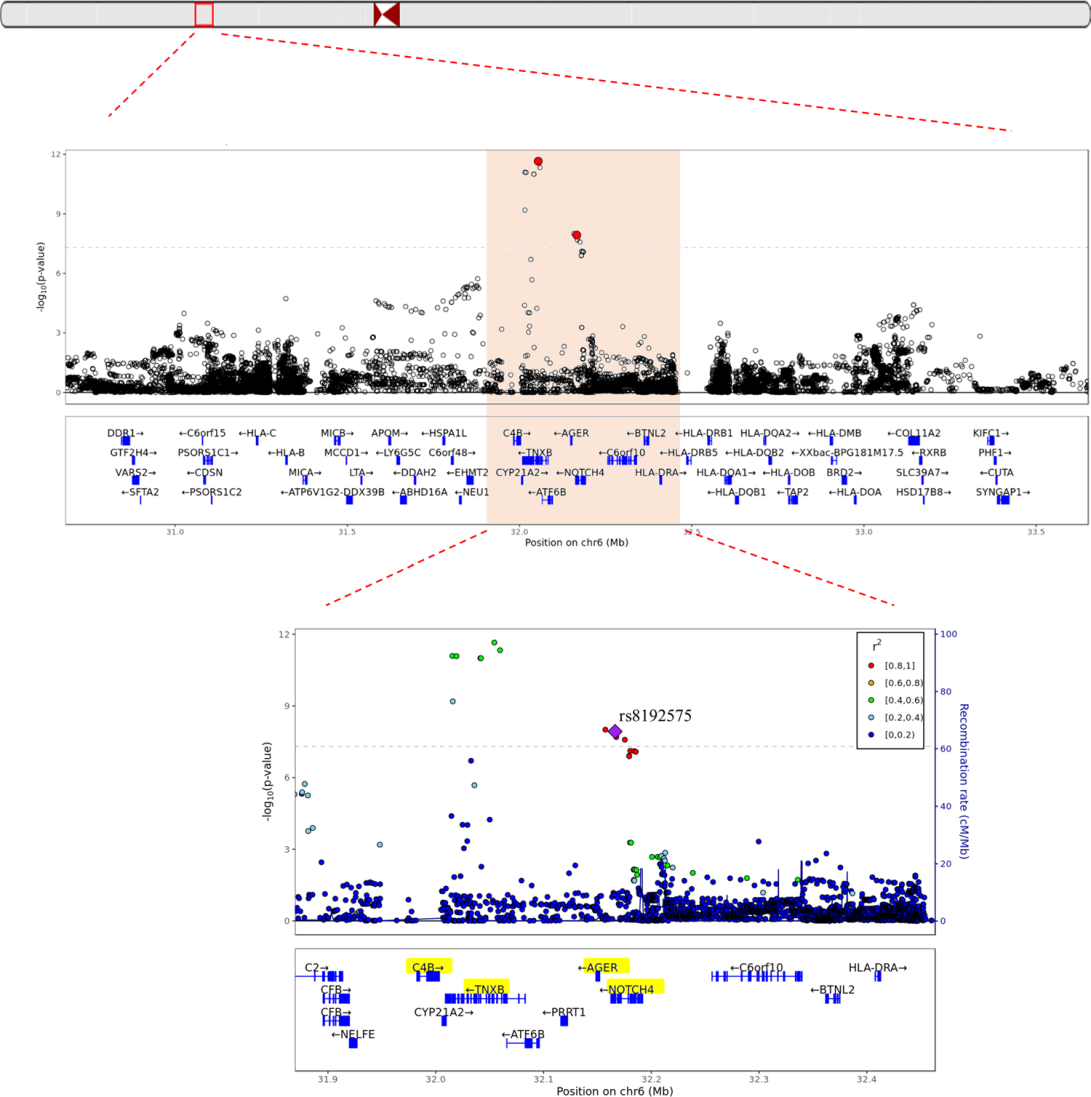
Genomic region on chromosome 6 near rs10947231 and rs8192575 Fig. 3 suggests that the rs10947231 and rs8192575 located at gene dense region. The plot was generated by software R version 3.6.1 (R Foundation for Statistical Computing; Vienna, Austria).

The GWIS of the FEV_1_/FVC ratio was conducted for the 7473 healthy individuals and 3,351,033 SNPs that passed the QC (Fig. 1). The clinical characteristics of the healthy individuals in the KARE cohort are shown in Table 1. The quantile–quantile plot in Fig. 2A shows that GWIS statistics retained the nominal significance level (variance inflation factor = 1.002). The Manhattan plot in Fig. 2B shows that 11 SNPs at 6p21 reached genome-wide significance levels. As shown in Supplementary Fig. 1, these 11 SNPs were distributed in two separate linkage disequilibrium (LD) block. The regional plot in Fig. 3 shows that the most significant SNPs were found near *TNXB*, with many other proximal genes. The most statistically significant result was obtained for rs10947231, an intronic SNP located in *TNXB*, with *P*_LR_ = 2.23 × 10^–12^ and corresponding *P*_SNP_ = 1.42 × 10^–10^ and *P*_INT_ = 0.84 (Table 2). Here, *P*_LR_ indicates the likelihood ratio test for ${\text{H}}_{0} :\beta_{SNP} = \beta_{SNP*pack - years} = 0{\text{ vs H}}_{1} : not {\text{H}}_{0}$.

**Table 1:** Descriptive statistics of healthy individuals Means of variables and their standard errors are calculated for continuous variables.

	KARE	GENIE	ARIRANG
Participants	7473	4413	513
Age (years)	52.0 ± 8.9	49.7 ± 6.8	59.0 ± 7.4
**Gender**			
Male	3310 (44.3%)	2507 (56.8%)	208 (40.5%)
Female	4163 (55.7%)	1906 (43.2%)	305 (59.5%)
Body mass index (kg/m^2^)	24.7 ± 3.1	23.3 ± 2.8	25.3 ± 3.2
Height (cm)	159.8 ± 8.7	165.6 ± 7.9	157.5 ± 8.5
**Smoking status**			
Non-smokers	4768 (63.8%)	2520 (57.1%)	369 (71.9%)
Former smokers	1140 (15.3%)	1380 (31.3%)	65 (12.7%)
Current smokers	1565 (20.9%)	513 (11.6%)	79 (15.4%)
Pack-years of smoking	22.8 ± 17.2	16.2 ± 24.1	27.5 ± 15.3
FEV_1_ (liters)	2.9 ± 0.7	3.0 ± 0.6	2.4 ± 0.6
FVC (liters)	3.6 ± 0.9	3.7 ± 0.8	3.1 ± 0.8
FEV_1_/FVC ratio	81.4 ± 5.4	81.7 ± 5.5	79.2 ± 5.1

**Table 2:** Results for rs10947231 and rs8192575 from discovery GWIS For FEV_1_/FVC, GWIS was performed on healthy individuals with 3,351,033 SNPs, and the genome-wide significant result are summarized. ${\upbeta }_{\mathrm{SNP}}$ and ${\upbeta }_{\mathrm{INT}}$ are the coefficients for the main SNP and interaction effects between SNP and pack-years of smoking, respectively. Overall effects indicate *P* values (P_LR_) for testing the null hypotheses ${\text{H}}_{0} :\beta_{SNP} = \beta_{SNP - pack\;years} = 0$ by F test.

	Data	Minor/Major alleles	MAF	*P* value for HWE	Missing rate	INFO^†^	${\upbeta }_{\mathrm{SNP}}$(SE)	*P*_SNP_	${\upbeta }_{\mathrm{INT}}$(SE)	*P*_INT_	Overall effects (P_LR_)
**rs10947231 Chromosome (BP) 6(32,054,346)**											
Discovery	KARE	A/C	0.165	0.767	0.015	0.983	0.835 (0.13)	1.42 × 10^–10^	0.001 (0.007)	0.8417	2.23 × 10^–12^
Replication	GENIE	A/C	0.18	0.902	0	0.999	0.178 (0.198)	0.3678	0.026 (0.014)	0.0684	0.0698
	ARIRANG	A/C	0.17	0.638	0.016	0.985	0.967 (0,473)	0.04151	− 0.056 (0.03)	0.0583	0.0714
**rs8192575 Chromosome (BP) 6(32,166,384)**											
Discovery	KARE	G/C	0.15	0.549	0.027	0.976	0.821 (0.136)	1.77 × 10^–9^	− 0.02 (0.008)	0.0165	1.18 × 10^–8^
Replication	GENIE	G/C	0.163	0.425	0	1	0.533 (0.21)	0.0113	− 0.031 (0.016)	0.0454	0.0173
	ARIRANG	G/C	0.176	0.357	0.006	0.996	0.702 (0.484)	0.1471	− 0.067 (0.027)	0.0131	0.0457

*BP* physical position (Based on hg19), *MAF* minor allele frequencies, *HWE* Hardy–Weinberg equilibrium, *SE* standard error.

^†^INFO is the imputation quality metric obtained from IMPUTE2.

**Figure 4:**
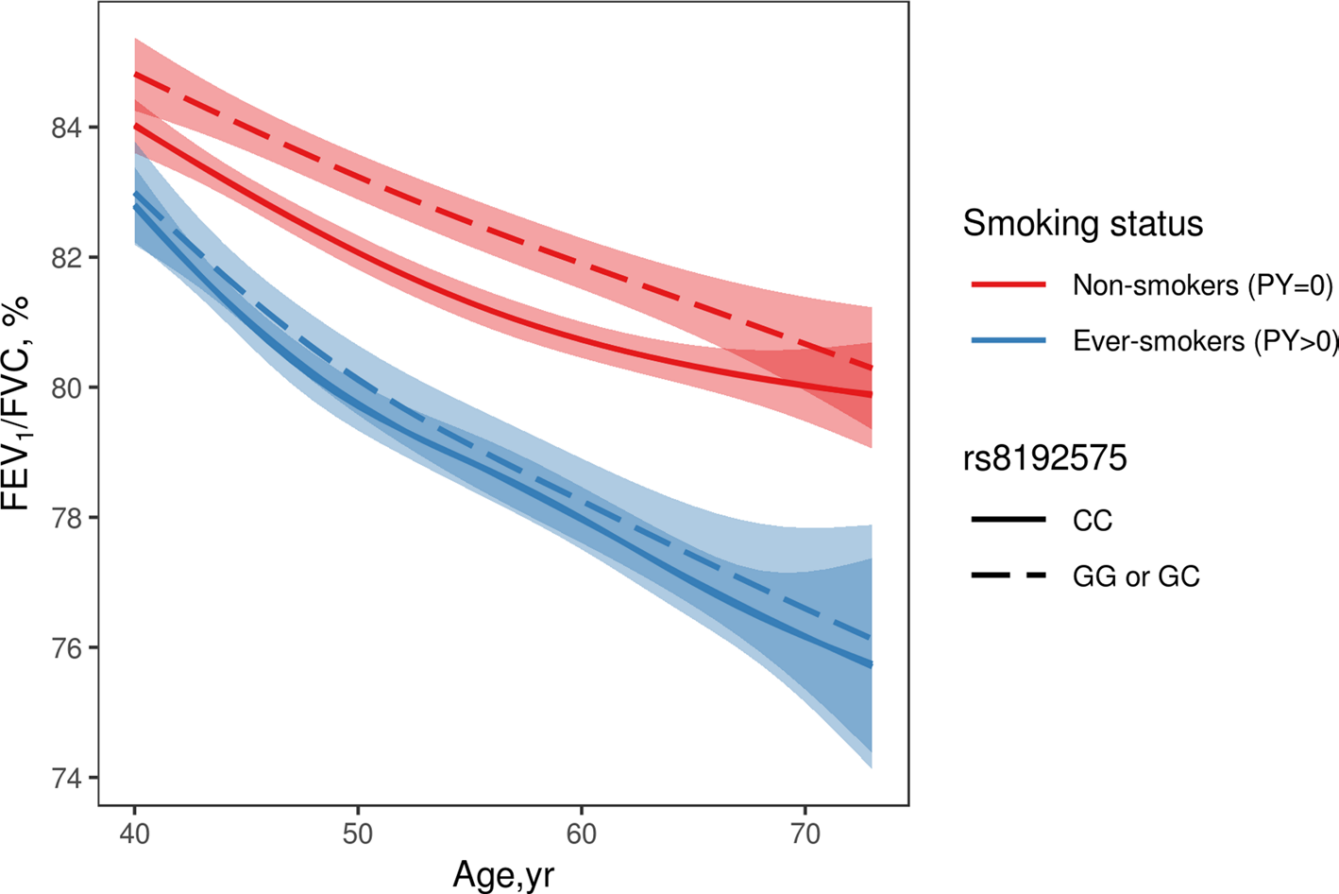
Declines of FEV_1_/FVC along age Changes in FEV_1_/FVC along age according to smoking status and rs8192575 were plotted by generalized additive models (GAM). Figure 4 suggests that smoking has a significant effect on FEV_1_/FVC ratios for healthy individuals. The plot was generated by software R version 3.6.1 (R Foundation for Statistical Computing; Vienna, Austria).

**Figure 5:**
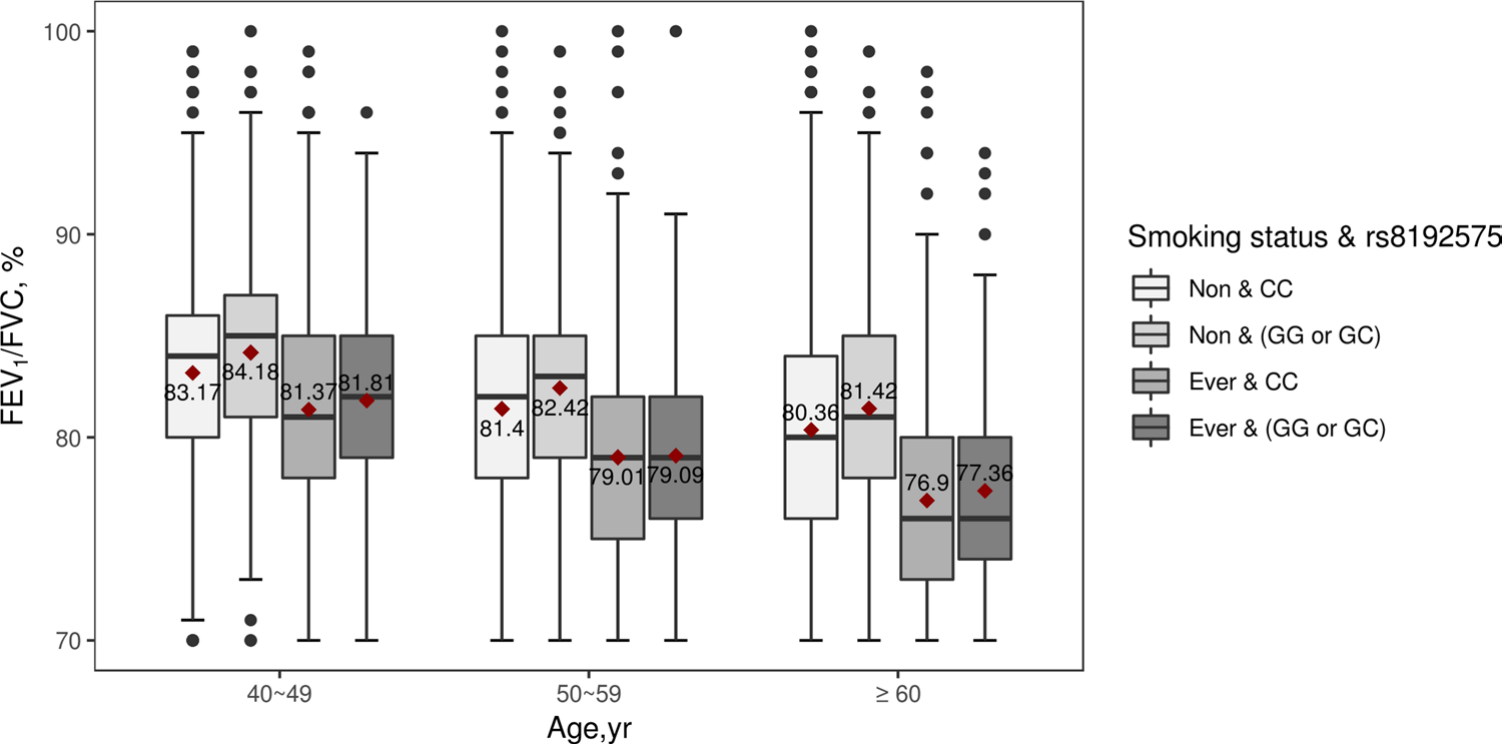
The boxplot of the FEV1/FVC grouped by rs8192575, smoking status, and age from KARE data We visualized the effects of rs819275 on FEV_1_/FVC according to smoking status and age. The plot represents the effects of rs8192575 for healthy individuals, and the allele G of rs8192575 has positive effects if healthy individuals have not smoked. The red diamond symbols represent mean value, which is displayed as a number. The plot was generated by software R version 3.6.1 (R Foundation for Statistical Computing; Vienna, Austria).

**Figure 6:**
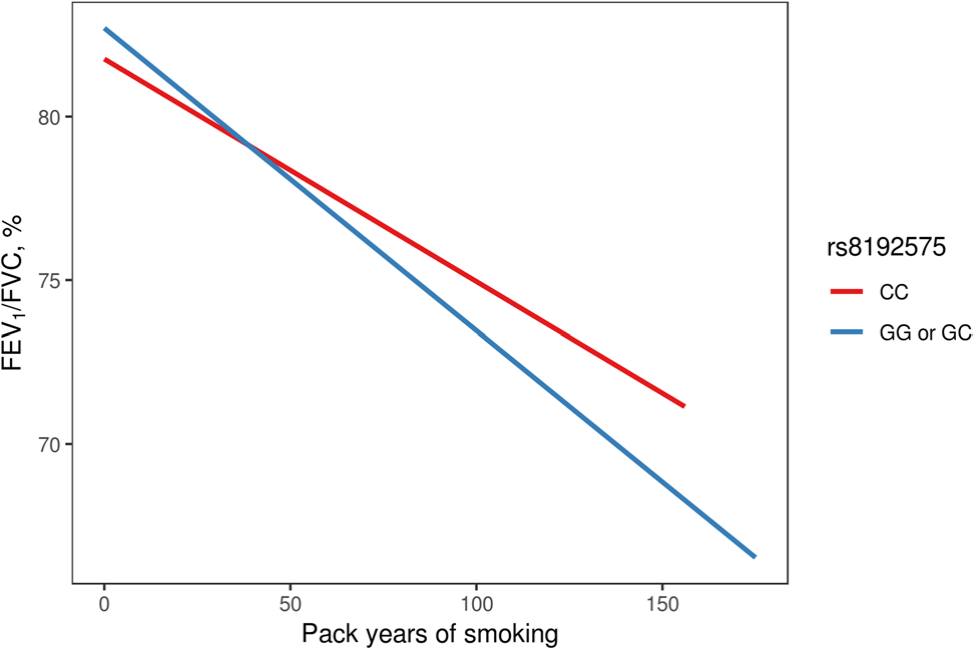
Estimated FEV1/FVC according to pack-years of smoking and rs8192575 The estimated FEV_1_/FVC ratio is generated by gene-by-smoking analysis using healthy individuals of KARE. Figure 6 implies that minor alleles G has negative impact in proportion to each increment of pack-years of smoking. The plot was generated by software R version 3.6.1 (R Foundation for Statistical Computing; Vienna, Austria).

The second genome-wide significant region was found near the *NOTCH4* intron. In the linkage disequilibrium (LD) block, the rs8192575 SNP showed a genome-wide significant overall effect. The *P*_LR_ was 1.18 × 10^–8^ (Table 2). The coefficients for the SNP and interaction effects were 0.821 and − 0.02, respectively (*P*_SNP_ = 1.77 × 10^–9^ and *P*_INT_ = 0.0165). These results indicated that if the genotype of rs8192575 is GG, the FEV_1_/FVC ratio tends to increase to approximately 0.821 × 2; however, for smokers, the FEV_1_/FVC ratios decrease to approximately − 0.02 × 2 per pack-year. Figure 4 indicates a significant difference in the FEV_1_/FVC ratios between non-smokers and smokers. Figure 5 presents box-plots in accordance with the smoking status, age, and genotypes of rs8192575. In healthy individuals, the FEV_1_/FVC ratios are consistently larger for non-smokers with genotypes GG and GC than for non-smokers with CC genotypes; however, for smokers, there are no significant differences in FEV_1_/FVC by allele G in rs8192575. Estimated FEV_1_/FVC ratios in accordance with the pack-years is shown in Fig. 6. In Fig. 6, the decreasing rate of FEV_1_/FVC is greater for individuals with genotypes GG and GC than genotypes CC. This indicates that the effect of allele G of rs8192575 is modified by pack-years of smoking.

Other nine significant SNPs were belonging in the same LD blocks with rs10947231 and rs8192575 (Supplementary Fig. 1), and the results are summarized in Supplementary Table 1.

### Validation analyses

The associations between rs10947231 and rs8192575 and the FEV_1_/FVC ratio were further assessed using the healthy individuals in GENIE and ARIRANG cohorts. Clinical characteristics of healthy individuals these datasets are presented in Table 1. Table 2 shows that rs10947231 overall effects were replicated in the GENIE and ARIRANG data at *P* < 0.1 (*P*_LR_ = 0.0698 for GENIE; *P*_LR_ = 0.0714 for ARIRANG). However the main SNP effects were not significant for GENIE data. Interestingly, for ARIRANG data, rs10947231 showed significant effects on both the SNP and interaction with *P*_SNP_ = 0.0415 and *P*_INT_ = 0.0583.

According to Table 2, the overall effect of rs8192575 was significant for GENIE and ARIRANG data (*P*_LR_ for GENIE = 0.0173 and *P*_LR_ for ARIRANG = 0.0457). For the GENIE data, both the main SNP and interaction effects were replicated at 0.05 significance levels (*P*_SNP_ = 0.0113 and *P*_INT_ = 0.0454), and their regression coefficients were 0.533 and -0.031, respectively. For the ARIRANG data, the interaction effect was replicated with -0.067 regression coefficients and 0.0131 *P* value. However, the main SNP effect was not significant (*P*_SNP_ = 0.1471). The results for the other nine significant SNPs are presented in Supplementary Table 2, and are similar to those found for rs10947231 and rs8192575.

### TAD, PrediXcan, and eQTL analyses

We considered chromatin TADs containing rs10947231 and rs8192575. TADs were defined using Hi-C to identify chromatin regions with physical contact. Most SNPs associated with human disease or other phenotypes may develop associations through interactions with regulatory elements of a coding gene within the SNP-bearing TAD^33^. Chromatin TAD analysis of lung tissue revealed that *TNXB*, *NOTCH4*, *AGER*, and *C4B* were in the same region (Supplementary Fig. 2). The most active interaction was observed in *NOTCH4*, followed by *AGER*, *TNXB*, and *C4B*, showing no significant differences.

PrediXcan analysis predicted susceptibility on the expression of chromosome 6 genes in lung tissue that regulate the FEV_1_/FVC ratio. The results, summarized in Table 3, evidenced *AGER* (*P* value = 8.59 × 10^–6^) as the gene most associated with the FEV_1_/FVC ratio. These results were significant at the conservative Bonferroni-adjusted 0.05 significance level (*P* value < 1.1 × 10^–4^).

**Table 3:** Result of PrediXcan for FEV_1_/FVC ratio on KARE using lung tissue prediction model The results for PrediXcan for the top 10 genes on chromosome 6 are summarized.

Gene	PrediXcan Beta	PrediXcan *P* value
AGER	− 2.797	8.59 × 10^–6^
HLA-S	− 0.833	4.60 × 10^–5^
C4B	− 0.736	0.0003
MDGA1-201	1.312	0.0012
HLA-J	1.065	0.0019
NKAPL	− 0.941	0.0042
HLA-DPA1	− 0.791	0.0052
HCG4B	0.716	0.0088
TPT1P4	1.522	0.0094
HLA-C	-0.549	0.0099

The eQTL of rs10947231 and rs8192575 were analyzed using GTEx (Supplementary Table 3). For rs10947231, no significant association between rs10947231 genotype and gene expression was found. For rs8192575, many eQTL genes, such as *NOTCH4*, *C4B*, and *AGER* were identified. Interestingly, *AGER* and *C4B* were differentially expressed in lung tissue, based on rs8192575. Thus, we further analyzed *AGER* and *C4B* expression using GTEx V7 data (Supplementary Fig. 3), and this revealed that *AGER* was upregulated in lung tissue, while *C4B* was expressed in adrenal glands and liver tissue. The numbers of transcripts per million kilobases for *C4B* and *AGER* in lung tissue were 5.53 and 1093.06, respectively.

In summary, it is unclear which gene, *AGER* or *C4B*, contributes to the significant effect of rs8192575 on the FEV_1_/FVC ratio. However, *AGER* might be a more promising candidate gene due to its higher expression levels in lung tissue compared to that of *C4B*.

### Independence between rs8192575 and smoking

Significant SNP-by-environment interactions are detected in the absence of true SNP-by-environment interactions if SNP and the environment are correlated. Thus, we investigated whether rs8192575 and smoking are independent, considering smoking status and pack-years as the smoking variables (Table 4). Supplementary Fig. 4 shows a boxplot of pack-years based on rs8192575; this SNP was not significantly associated with pack-years of smoking. Therefore, the statistical significance of the rs8192575-by-smoking interaction may indicate biological interactions, especially among subjects with normal lung function, and this could be true causal effects.

**Table 4:** Independence test between rs8192575 and smoking variables We tested gene-smoking dependency. The association between rs8192575 and pack-years of smoking are analyzed by linear regression, and the association between rs8192575 and smoking status are analyzed by logistic regression. For KARE and ARIRANG data the smoking status indicates non- and ever-smokers. For GENIE data smoking status indicates non- and current smokers. *P *value greater than 0.05 suggests that there was no dependency between rs8192575 and smoking variables.

DATA	*P *value from healthy individuals
**Response variable: Pack-years of smoking**	
KARE	0.659
GENIE	0.3294
ARIRANG	0.8955
**Response variable: Smoking status (never- and ever-smokers for KARE, ARIRANG; never- and current-smokers for GENIE)**	
KARE	0.7734
GENIE	0.2881
ARIRANG	0.7462

## Discussion

The present GWIS including healthy individuals from the KARE dataset found that rs10947231 and rs8192575, located on chromosome 6 (6p21), were significantly associated with the FEV_1_/FVC ratio in healthy individuals. Furthermore, rs8192575 showed significant interaction effects with the smoking variable pack-years of smoking. These associations was further investigated using data from two other Korean cohorts (GENIE and ARIRANG). For rs10947231 and rs8192575, both cohorts showed significant overall effects (*P* < 0.1) and significant interaction effects with pack-years. The most significant findings were found in the introns of *TNXB* (rs10947231) and *NOTCH4* (rs8192575). Region 6p21 is gene-dense, including genes *TNXB*, *PPT2*, *C4B*, *NOTCH4*, and *AGER*, among others. We conducted TAD, PrediXcan, and eQTL analyses to confirm the strongly associated genes. We confirmed that rs8192575 was strongly associated with *AGER* in lung tissues, and *AGER* has been previously described as susceptible for lung function and COPD. The rs10947231 does not have any eQTL genes. Its MAF in Europeans were 0.058 and 0.002 with 1000 Genome and gnomAD^34^, respectively, and its low MAF may induce non-significance of its eQTL analysis. Considering that rs10947231 is in the TADs block of *AGER* region, and DNA sequences within TAD interact more frequently with each other than those outside TAD, there are some possibilities of significant association between *AGER* and rs10947231.

*AGER* is a protein coding gene which encodes RAGE belonging to the immunoglobulin superfamily and cell-surface receptor^35^. The RAGE has been extensively studied, and it significantly contributes to lung development and to maintain adult lung homeostasis, as evidenced by its upregulation on the membrane and cytoplasm of both Type 1 alveolar cells and macrophages^36^. A recent study suggested that RAGE upregulation during lung development inhibits alveolar morphogenesis and induces significant changes in morphometric parameters, including a reduction in airspace and an increase in alveolar duct size^37^. Lee et al.^38^ reported that the blockade of RAGE is significantly associated with decreased pulmonary inflammation and inhibits the activation of damage-associated molecular patterns in mice exposed to tobacco smoke. Indeed, RAGE expression increased after exposure to tobacco smoke and in COPD patients^39^ suggesting that RAGE suppression protects against COPD. The eQTL analysis revealed that the minor allele G of rs8192575 was associated with lower *AGER* expression in lung tissue; thus, *AGER* may be downregulated in individuals with a larger number of G alleles. Figures 4 and 5 show that participants with G alleles also tended to have a greater FEV_1_/FVC ratio, suggesting that *AGER* is associated with rs8192575 (This result is consistent with the previous report that RAGE suppression protect against COPD). The circulating soluble RAGE (sRAGE), acting as decoy receptors, has been robustly demonstrated that low sRAGE levels are associated with advanced COPD and lung function decline, which may be counterintuitive with our results. One possible explanation is that the effect of genetic variants on sRAGE protein levels is affected by environmental exposure, which indicates gene × smoking interaction effects. Another possibility is that genetic variants that promote the association between sRAGE and lung disease susceptibility have different mechanism^40^.

Nonetheless, this study has some limitations. First, the 6p21 gene-dense region and *C4B* might have affected the eQTL and PrediXcan results. Gene *C4B* is a product of complement C4 activated in the early stage of the mannose-binding lectin pathway^41^, and some studies have reported that *C4B* is associated with tissue damage in pulmonary tuberculosis patients^42,43^. However, *C4B* was not upregulated in lung tissues (Supplementary Fig. 3), and its role in pulmonary function is still lesser known than that of *AGER*. Thus, we concluded *AGER* is a more promising candidate gene for COPD. Second, the most significant SNP, rs10947231, showed *P* = 1.42 × 10^–10^ for its main effects in the KARE dataset, but only the ARIRANG data replicated this significance. Because rs10947231 was not directly associated with *AGER*, allelic and locus heterogeneities could be possible reasons for the failure to replicate such significant effects in the GENIE dataset. Third, the rs2070600 located in *AGER*, previously reported to be associated with the FEV_1_/FVC ratio in the European population and associated with COPD, emphysema, and sRAGE levels, was not examined in our study. This SNP was excluded in our discovery GWIS during genotype QC and it did not show statistically significant interaction effects (*P*_LR_ = 1.673 × 10^–15^, *P*_SNP_ = 1.87 × 10^–13^, *P*_INT_ = 0.984), potentially due to differences in the study population. These differences may be attributable to differences in genetic ancestry and LD structure among populations. Fourth, the overall and main SNP effects of rs8192575 were significant throughout the genome (significance level = 5 × 10^–8^). However, SNP-by-smoking interactions were significant at a relatively high significance level, i.e., at 0.05. Analyses of gene-by-environment interactions often present numerous false-negative results, concurrent with the present findings. Hence, a larger sample is necessary to obtain genome-wide significant results to analyze gene-by-environment interactions. Fifth, our functional analyses (eQTL, TAD, and PrediXcan) have some limitations. For eQTL and TAD, the relevance of their results depends on tissue type. COPD and FEV_1_/FVC are particularly related to lung tissue and it was chosen. However it is still possible that the other tissue can be a better choice^44^. Furthermore, the prediction model of PrediXcan was built by European but it was applied to Korean. In such case, the prediction accuracy can become worse^45,46^.

In conclusion, we identified genome-wide significant effects at the 6p21 region using the FEV_1_/FVC of healthy individuals, and rs8192575 showed significant interaction effects with smoking. Indeed, 6p21 is a gene-dense region, as characterized by previous GWAS.^7,8,47^ However, significant results were obtained with healthy individuals, and evidence regarding its significant interaction effects with smoking were found in Korean cohort data. The MAF of rs8192575 for the European population was 0.083 with 1000G European samples. Therefore, the lack of significant results so far might be due to low allele frequencies. However, for Koreans, the MAF was relatively high (0.15 to 0.18), and rs8192575 seems to have a significant effect on the Korean population. We expect that these results potentially provide insights into COPD pathogenesis and on the effect of smoking on lung function, concurrent with previous GWAS and biological reports.

## Supplementary information

Supplementary information

## Data Availability

Genotype and clinical data for KARE and ARIRANG can be downloaded from https://koreabiobank.re.kr followed by an approval process from Korean NIH. For more detail information for approval process, please contact to biobank@korea.kr. All type data for GENIE cohort is available on request at https://en-healthcare.snuh.org/HPEACEstudy. In addition, all the data analyzed in this article was utilized in previously published articles (KARE: Cho et al. ^18^, ARIRANG: Huh et al.^21^, GENIE: Lee et al.^19^.
