# Supplementary material for: Effect of 6p21 region on lung function is modified by smoking: a genome-wide interaction study
Source: Sci Rep. 2020 Aug 4;10:13075. doi: 10.1038/s41598-020-70092-0 (PMC7403370; doi:10.1038/s41598-020-70092-0)

Web-based Supplementary Materials for "**Effect 6p21 region on lung function is modified by smoking: a genome-wide interaction study**"

Boram Park[1], Jaehoon An[1], Wonji Kim[2], Hae Yeon Kang[3], Sang Baek Koh[4], Bermseok Oh[5], Keum Ji Jung[6], Sun Ha Jee[6], Woo Jin Kim[7], Michael H. Cho[8,9], Edwin K. Silverman[8,9], Taesung Park [2,10*], Sungho Won[1,2,11*]

[1]Department of public health sciences, Seoul national university, Seoul, South Korea.

[2]Interdisciplinary Program of Bioinformatics, Seoul National University, Seoul, South Korea

[3]Department of Internal Medicine, Healthcare Research Institute, Seoul National University Hospital Healthcare System Gangnam Center, Seoul, South Korea.

[4]Department of Preventive Medicine, Yonsei University Wonju College of Medicine, Wonju, South Korea.

[5]Department of Biochemistry and Molecular Biology, School of Medicine, Kyung Hee University, Seoul, South Korea

[6]Institute for Health Promotion, Graduate School of Public Health, Yonsei University, Seoul, South Korea

[7]Department of Internal Medicine and Environmental Health Center, Kangwon National University Hospital, School of Medicine, Kangwon University Chuncheon, South Korea

[8]Channing Division of Network Medicine, Department of Medicine, Brigham and Women's Hospital and Harvard Medical School, Boston, Massachusetts, United States of America.

[9]Division of Pulmonary and Critical Care Medicine, Department of Medicine, Brigham and

Women's Hospital, Harvard Medical School, Boston, Massachusetts, United States of America.

[10]Department of Statistics, Seoul National University, Seoul, South Korea

[11]Institute of Health and Environment, Seoul National University, Seoul, South Korea.

Corresponding Authors:

Sungho Won, Department of Public Health Science, Seoul National University

1 Kwanak-ro Kwanak-gu Seoul 151-742 Korea

(Email) sunghow@gmail.com, (Tel) +82-2-880-2714, (Fax) +82-303-0942-2714

Taesung Park, Department of Statistics, Seoul National University

1 Kwanak-ro Kwanak-gu Seoul 151-742 Korea

(E-mail) tspark@stats.snu.ac.kr, (Tel) +82-2-880-8924, (Fax) +82-2-888-6693

Supplementary Table 1 Results of GWIS on normal individuals with KARE data β_{SNP} and β_{INT} are the coefficients for the main SNP and interaction effects between SNP and pack-years of smoking, respectively. Overall effects indicate P-values(P_{LR}) for testing the null hypotheses $H_0: \beta_{SNP} = \beta_{SNP-pack\ years} = 0$ by F test.

SNP	CHR	BP	Minor/Major Alleles	MAF	P-value for HWE	Missing rate	INFO†	β_{SNP} (SE)	P_{SNP}	β_{INT} (SE)	P_{INT}	Overall effects (P_{LR})
**rs10947231**	6	32054346	A/C	0.165	0.767	0.015	0.983	0.835(0.13)	1.42×10^{-10}	0.001(0.007)	0.8417	2.23×10^{-12}
rs9391733	6	32059674	G/C	0.164	0.734	0.019	0.982	0.829(0.13)	2.22×10^{-10}	0.001(0.007)	0.8785	4.66×10^{-12}
rs118104272	6	32015428	T/C	0.164	0.641	0.011	0.985	0.815(0.13)	3.73×10^{-10}	0.001(0.007)	0.8571	8.05×10^{-12}
rs74290528	6	32019235	C/A	0.164	0.672	0.011	0.986	0.813(0.13)	4.09×10^{-10}	0.001(0.007)	0.8390	8.26×10^{-12}
rs9368704	6	32041373	A/G	0.164	0.611	0.01	0.992	0.808(0.13)	5.11×10^{-10}	0.002(0.007)	0.8183	1.00×10^{-11}
rs9366789	6	32041931	G/C	0.164	0.611	0.01	0.992	0.808(0.13)	5.11×10^{-10}	0.002(0.007)	0.8183	1.00×10^{-11}
rs2021783	6	32044851	T/C	0.164	0.352	0	1	0.789(0.129)	9.44×10^{-10}	0.002(0.007)	0.7821	1.78×10^{-11}
rs78125577	6	32015796	G/A	0.218	0.43	0.022	0.98	0.672(0.119)	1.65×10^{-8}	0.002(0.006)	0.7812	6.46×10^{-10}
**rs8192575**	6	32166384	G/C	0.15	0.549	0.027	0.976	0.821(0.136)	1.77×10^{-9}	-0.02(0.008)	0.0165	1.18×10^{-8}
rs9378121	6	32167513	T/C	0.15	0.581	0.027	0.977	0.808(0.136)	3.08×10^{-9}	-0.019(0.008)	0.0188	2.02×10^{-8}
rs200391889	6	32175415	A/C	0.15	0.783	0.024	0.981	0.799(0.136)	3.91×10^{-9}	-0.019(0.008)	0.0180	2.60×10^{-8}

-Definition of Abbreviations: BP = physical position (Based on hg19); MAF = minor allele frequencies; HWE = Hardy-Weinberg equilibrium; SE = standard error

†INFO is the imputation quality metric obtained from IMPUTE2;

Supplementary Table 2 Validation analyses on the most significant SNPs from discovery GWIS β_{SNP} and β_{INT} are the coefficients for the main SNP and interaction effects between SNP and pack-years of smoking, respectively. Overall effects indicate P-values(P_{LR}) for testing the null hypotheses $H_0: \beta_{SNP} = \beta_{SNP-pack\ years} = 0$ by F test.

(A) Results from GENIE data

SNP	CHR	BP	Minor/Major Alleles	MAF	P-value for HWE	Missing rate	INFO†	β_{SNP} (SE)	P_{SNP}	β_{INT} (SE)	P_{INT}	Overall effects (P_{LR})
**rs10947231**	6	32054346	A/C	0.18	0.902	0	0.999	0.178(0.198)	0.3678	0.026(0.014)	0.0684	0.0698
rs9391733	6	32059674	G/C	0.179	0.951	0.004	0.998	0.164(0.198)	0.4086	0.026(0.014)	0.0678	0.0769
rs118104272	6	32015428	T/C	0.18	0.854	0.001	0.997	0.197(0.198)	0.3207	0.025(0.014)	0.0813	0.0721
rs74290528	6	32019235	C/A	0.18	0.806	0	0.998	0.198(0.198)	0.3170	0.025(0.014)	0.0812	0.0712
rs9368704	6	32041373	A/G	0.18	0.854	0	0.999	0.182(0.198)	0.3568	0.026(0.014)	0.0680	0.0673
rs9366789	6	32041931	G/C	0.18	0.854	0	0.999	0.182(0.198)	0.3568	0.026(0.014)	0.0680	0.0673
rs2021783	6	32044851	T/C	0.17	0.273	0	1	0.191(0.199)	0.3361	0.028(0.014)	0.0422	0.0396
rs78125577	6	32015796	G/A	0.234	0.389	0.003	0.997	0.237(0.181)	0.1914	0.012(0.014)	0.4133	0.2035
**rs8192575**	6	32166384	G/C	0.163	0.425	0	1	0.533(0.21)	0.0113	-0.031(0.016)	0.0454	0.0173
rs9378121	6	32167513	T/C	0.163	0.319	0	1	0.565(0.21)	0.0072	-0.036(0.016)	0.0258	0.0088
rs200391889	6	32175415	A/C	0.163	0.352	0	0.999	0.562(0.21)	0.0075	-0.037(0.016)	0.0235	0.0086

(B) Results from ARIRANG data

SNP	CHR	BP	Minor/Major Alleles	MAF	P-value for HWE	Missing rate	INFO†	β_{SNP} (SE)	P_{SNP}	β_{INT} (SE)	P_{INT}	Overall effects (P_{LR})
**rs10947231**	6	32054346	A/C	0.17	0.638	0.016	0.985	0.967(0.473)	0.0415	-0.056(0.03)	0.0583	0.0714
rs9391733	6	32059674	G/C	0.171	0.639	0.018	0.984	0.966(0.474)	0.0420	-0.056(0.03)	0.0588	0.0723
rs118104272	6	32015428	T/C	0.171	0.639	0.018	0.98	0.999(0.473)	0.0351	-0.057(0.03)	0.0541	0.0620
rs74290528	6	32019235	C/A	0.171	0.639	0.018	0.981	0.999(0.473)	0.0351	-0.057(0.03)	0.0541	0.0620
rs9368704	6	32041373	A/G	0.17	0.638	0.016	0.986	0.967(0.473)	0.0415	-0.056(0.03)	0.0583	0.0714
rs9366789	6	32041931	G/C	0.17	0.638	0.016	0.986	0.967(0.473)	0.0415	-0.056(0.03)	0.0583	0.0714
rs2021783	6	32044851	T/C	0.176	0.761	0	1	0.88(0.467)	0.0602	-0.052(0.029)	0.0766	0.1046
rs78125577	6	32015796	G/A	0.212	0.494	0.066	0.959	0.9(0.446)	0.0442	-0.03(0.025)	0.2311	0.1257
**rs8192575**	6	32166384	G/C	0.176	0.357	0.006	0.996	0.702(0.484)	0.1471	-0.067(0.027)	0.0131	0.0457
rs9378121	6	32167513	T/C	0.176	0.357	0.006	0.996	0.702(0.484)	0.1471	-0.067(0.027)	0.0131	0.0457
rs200391889	6	32175415	A/C	0.178	0.222	0.019	0.984	0.612(0.483)	0.2051	-0.066(0.027)	0.0145	0.0499

-Definition of Abbreviations: MAF = minor allele frequencies; HWE = Hardy-Weinberg equilibrium; SE = standard error

†INFO is the imputation quality metric obtained from IMPUTE2

Supplementary Table 3 Results of eQTL significantly affected by rs8192575

Gene	Tissue	Standardized effect	P-value
HLA-C	Muscle-Skeletal	-0.57	4.8×10^{-7}
CSNK2B	Brain - Nucleus accumbens	-0.82	1.8×10^{-6}
NOTCH4	Muscle-Skeletal	0.38	2.3×10^{-6}
**C4B**	**Lung**	**-0.50**	$\mathbf{3.0\times10^{-6}}$
ATF6B	Pancreas	0.65	1.5×10^{-5}
DDX39B	Whole Blood	-0.35	2.1×10^{-5}
C4B	Adrenal Gland	-0.79	2.3×10^{-5}
ATF6B	Heart - Left Ventricle	0.62	3.1×10^{-5}
**AGER**	**Lung**	**-0.22**	$\mathbf{4.5\times10^{-5}}$
C4A	Thyroid	0.44	5.8×10^{-5}
LY6G5B	Muscle-Skeletal	-0.22	6.8×10^{-5}
ATF6B	Nerve - Tibial	0.35	7.8×10^{-5}
CYP21A1P	Nerve - Tibial	0.58	8.6×10^{-5}
HLA-C	Skin - Sun Exposed	-0.43	1.2×10^{-4}

Supplementary Figure 1 Linkage disequilibrium among the eleven most significant SNPs
Graph shows the LD plot generated with Haploview software and r^2 values were used.

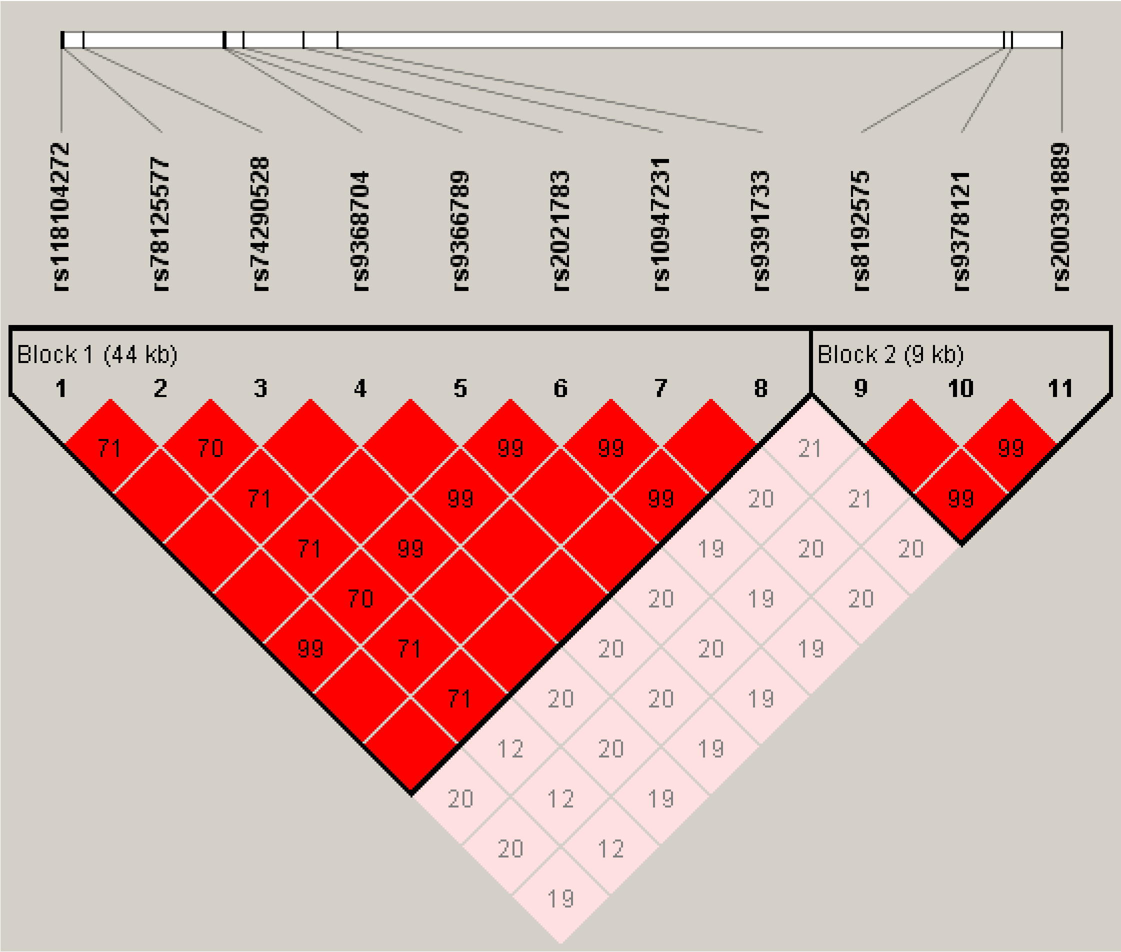

Supplementary Figure 2 Hi-C heatmap of TADs defined in lung cells The heatmap shows the topologically associated domains (TADs) defined by Hi-C analysis for genomic region of 6p21. From heatmap, it can be seen that *NOTCH4*, *AGER*, and *C4B* are located in the same TAD block of rs10947231 and rs8192575. As a deeper red color indicates the higher probability of interaction between the two regions, rs8192575 would have the most active interaction in the order *NOTCH4*, *AGER*, and *C4B*.

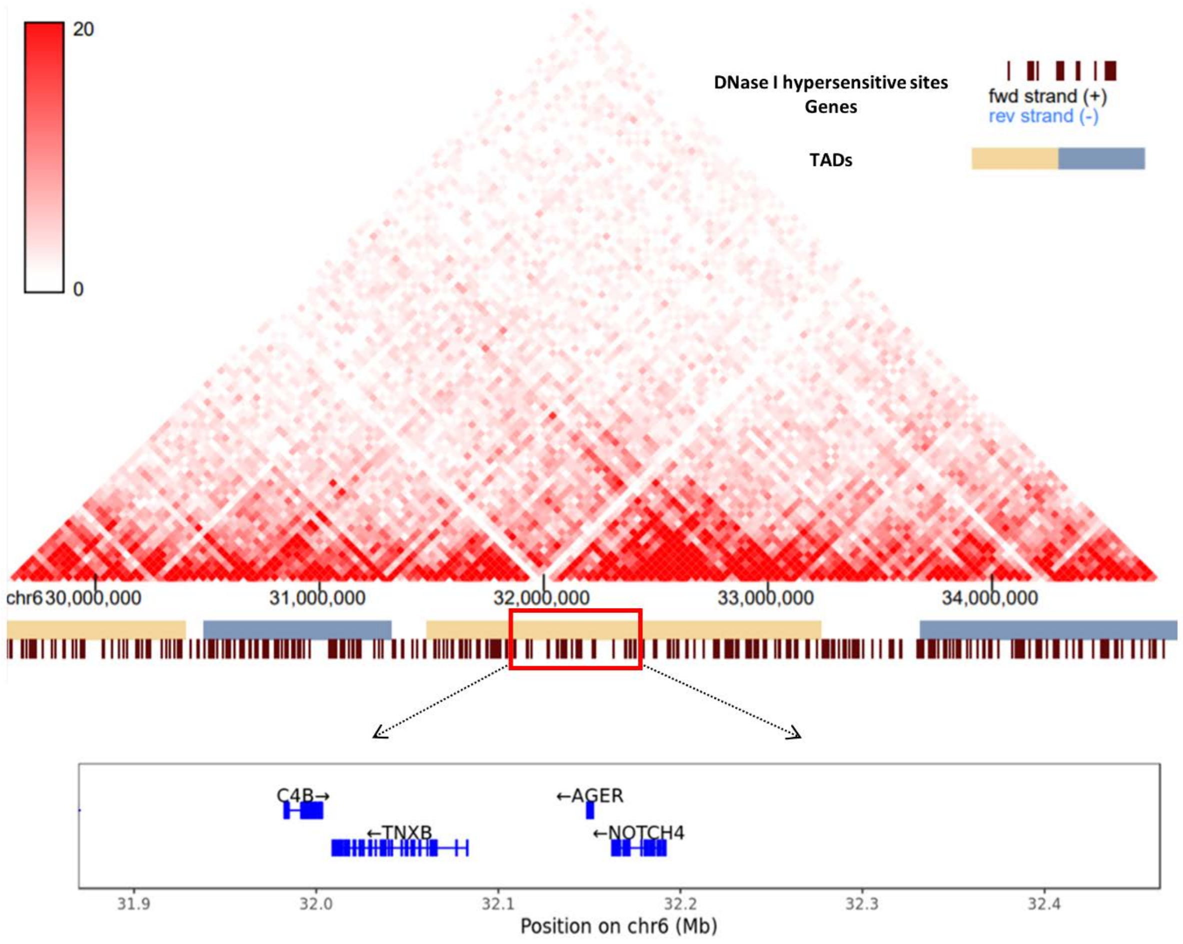

Supplementary Figure 3 Gene expression for *AGER* and *C4B* The exon-level expressions of AGER and C4B across all tissues are summarized. Plots were generated from GTEx portal. *AGER* and *C4B* shows 1093.06 and 5.53 transcripts per kilobase million in lung tissues, respectively.

AGER

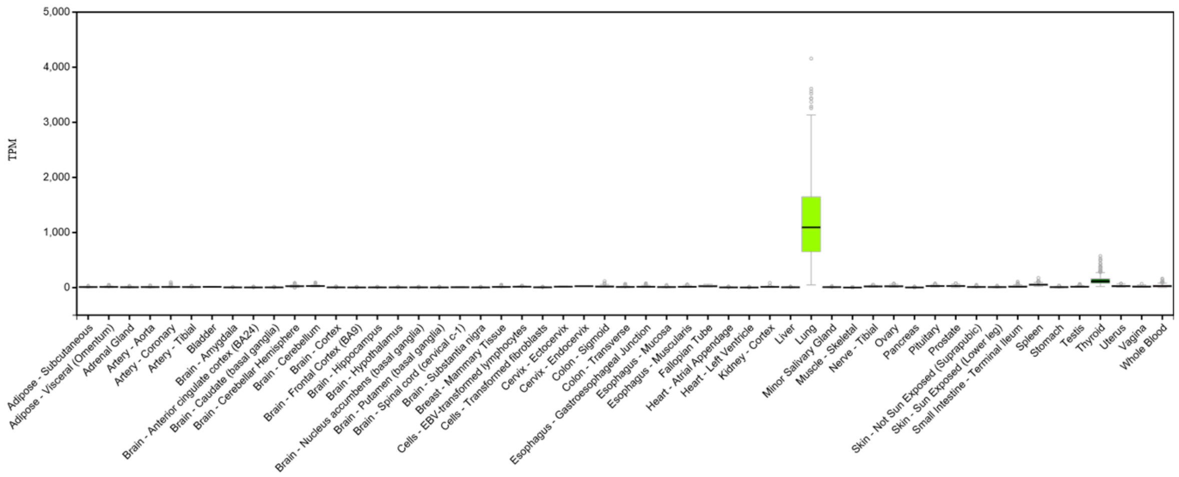

C4B

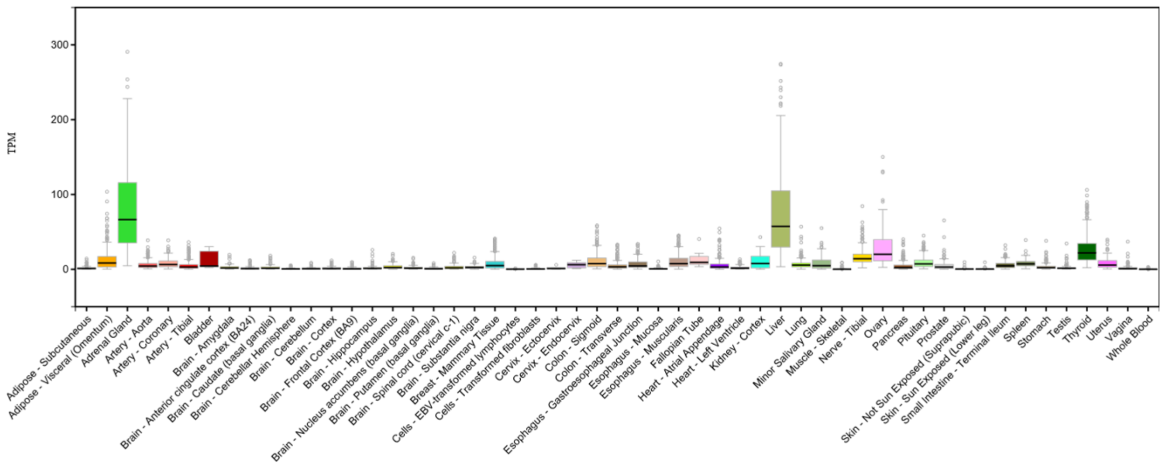

Supplementary Figure 4 The boxplot of the pack-years of smoking grouped by rs8192575
We visualized the association between rs8192575 and pack-years of smoking for each cohort. This suggest that the pack-years of smoking and the genotypes of rs8192575 are not related. The red diamond symbols represent mean value, which is displayed as a number.

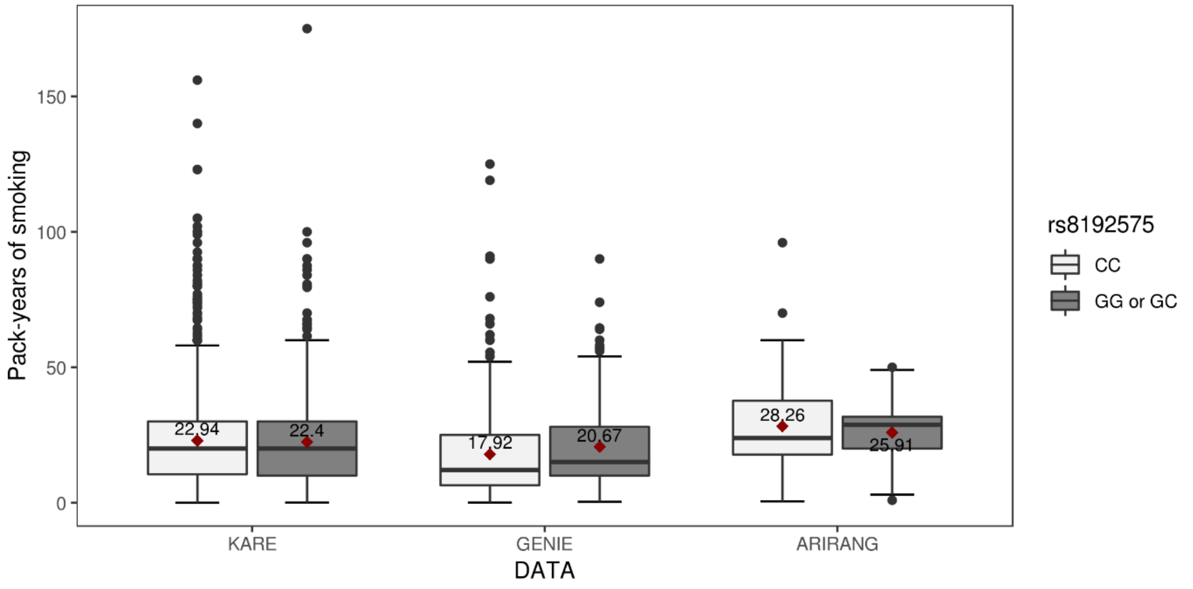

Supplement: Supplementary file 1 — Supplementary information [file 41598_2020_70092_MOESM1_ESM.pdf]
